# Expert consensus on odontogenic maxillary sinusitis multi-disciplinary treatment

**DOI:** 10.1038/s41368-024-00278-z

**Published:** 2024-02-01

**Authors:** Jiang Lin, Chengshuo Wang, Xiangdong Wang, Faming Chen, Wei Zhang, Hongchen Sun, Fuhua Yan, Yaping Pan, Dongdong Zhu, Qintai Yang, Shaohua Ge, Yao Sun, Kuiji Wang, Yuan Zhang, Mu Xian, Ming Zheng, Anchun Mo, Xin Xu, Hanguo Wang, Xuedong Zhou, Luo Zhang

**Affiliations:** 1 Department of Stomatology, Beijing TongRen Hospital, Capital Medical University, Beijing, China; 2 Department of Otolaryngology, Head and Neck Surgery, Beijing TongRen Hospital, Capital Medical University, Beijing, China; 3 Beijing Key Laboratory of Nasal Diseases, Beijing Institute of Otolaryngology, Beijing, China; 4 Department of Allergy, Beijing TongRen Hospital, Capital Medical University, Beijing, China; 5 State Key Laboratory of Oral & Maxillofacial Reconstruction and Regeneration, National Clinical Research Center for Oral Diseases, Shanxi International Joint Research Center for Oral Diseases, Department of Periodontology, School of Stomatology, The Fourth Military Medical University, Xi’ an, China; 6 Department of Oral and Maxillofacial Surgery, Peking University School and Hospital of Stomatology, National Center of Stomatology, National Clinical Research Center for Oral Diseases, Beijing, China; 7 Department of Oral &Maxillofacial Pathology, School and Hospital of Stomatology, Jilin University, Jilin, China; 8 Department of Periodontology, Nanjing Stomatological Hospital, Medical School of Nanjing University, Nanjing, China; 9 Department of Periodontics, School and Hospital of Stomatology, China Medical University, Shenyang, China; 10 Department of Otorhinolaryngology Head and Neck Surgery, China-Japan Union Hospital of Jilin University, Changchun, China; 11 Department of Otolaryngology, Head and Neck Surgery, The Third Affiliated Hospital, Sun Yat-sen University, Guangzhou, China; 12 Department of Periodontology, School and Hospital of Stomatology, Cheeloo College of Medicine, Shandong University & Shandong Key Laboratory of Oral Tissue Regeneration, Shandong Engineering Research Center of Dental Materials and Oral Tissue Regeneration, Shandong Provincial Clinical Research Center for Oral Diseases, Jinan, China; 13 Department of Implantology, Stomatological Hospital and Dental School of Tongji University, Shanghai Engineering Research Center of Tooth Restoration and Regeneration, Shanghai, China; 14 Research Unit of Diagnosis and Treatment of Chronic Nasal Diseases, Chinese Academy of Medical Sciences, Beijing, China; 15 State Key Laboratory of Oral Diseases & National Center for Stomatology & National Clinical Research Center for Oral Diseases & Department of Oral Implantology, West China Hospital of Stomatology, Sichuan University, Chengdu, China; 16 State Key Laboratory of Oral Diseases & National Center for Stomatology & National Clinical Research Center for Oral Diseases & Department of Cariology and Endodontics, West China Hospital of Stomatology, Sichuan University, Chengdu, China; 17 State Key Laboratory of Oral & Maxillofacial Reconstruction and Regeneration, National Clinical Research Center for Oral Diseases, Shaanxi Key Laboratory of Oral Diseases, Department of Operative Dentistry & Endodontics, School of Stomatology, The Fourth Military Medical University, Xi’an, China

**Keywords:** Oral diseases, Oral manifestations

## Abstract

Odontogenic maxillary sinusitis (OMS) is a subtype of maxillary sinusitis (MS). It is actually inflammation of the maxillary sinus that secondary to adjacent infectious maxillary dental lesion. Due to the lack of unique clinical features, OMS is difficult to distinguish from other types of rhinosinusitis. Besides, the characteristic infectious pathogeny of OMS makes it is resistant to conventional therapies of rhinosinusitis. Its current diagnosis and treatment are thus facing great difficulties. The multi-disciplinary cooperation between otolaryngologists and dentists is absolutely urgent to settle these questions and to acquire standardized diagnostic and treatment regimen for OMS. However, this disease has actually received little attention and has been underrepresented by relatively low publication volume and quality. Based on systematically reviewed literature and practical experiences of expert members, our consensus focuses on characteristics, symptoms, classification and diagnosis of OMS, and further put forward multi-disciplinary treatment decisions for OMS, as well as the common treatment complications and relative managements. This consensus aims to increase attention to OMS, and optimize the clinical diagnosis and decision-making of OMS, which finally provides evidence-based options for OMS clinical management.

## Introduction

Odontogenic maxillary sinusitis (OMS)^1^ or odontogenic sinusitis is inflammation of the maxillary sinus (MS), which is a consequence of lesions from the neighboring maxillary teeth or a result of iatrogenic damage during dental interventions.^2,3^ With its definite oral infectious pathogeny, OMS is distinct from other types of rhinosinusitis and requires a unique diagnostic and treatment regimen.^4^

The population-based incidence of OMS is not clear. In a retrospective research, a cohort of 385 subjects was evaluated by Wuokko-Landén et al., and they suggested that about 15% of acute rhinosinusitis may be odontogenic.^5^ Approximately one-quarter of chronic MS cases could be attributed to dental pathologies.^6^ Based on the computed tomography (CT) images of 130−190 patients from different studies, OMS cases was found to account for 45%−72% of cases with unilateral maxillary sinus opacification.^7–10^ A meta-analysis included 31studies has revealed that, on the basis of CT imaging, the aggregated prevalence of OMS was found to be 51% for each maxillary sinus and 50% on a per-patient basis.^11^ OMS, affecting males and females almost equally, commonly presents unilaterally.^3^ Although most OMS cases are chronic, they can also present acutely, and even spread to extrasinus orbital, intracranial, or parapharyngeal area.^12–14^ Besides, OMS can also present with concomitant fungal ball.

Appropriate dental and nasal treatment should be combined undoubtedly in OMS management. Although OMS is a curable disease with good prognosis, several issues regarding its management need to be solved urgently, including (a) the lack of attention, (b) the lack of proper diagnostic criteria, and (c)the lack of standardized management guideline. Firstly, comparing to other phenotypes of rhinosinusitis, OMS has received less attention, and is underrepresented by relatively low publication volume and quality. OMS-related literature only constituted about 1% of that of sinusitis over the last two decades.^15^ Moreover, during the last 30 years, over 90% of the published studies per decade have been ranked to level 4−5 evidence.^15^ Secondly, OMS tends to be missed diagnosed or misdiagnosed. Some OMS cases caused by apical periodontitis (AP) cannot be identified on CT scans.^16,17^ Some OMS cases were even found to be asymptomatic with regard to symptoms of dental and maxillary sinus.^12^ It is suggested that otorhinolaryngologists ought to direct patients exhibiting unilateral opacification of the maxillary sinus to dental experts for an evaluation of potential dental issues, and this advisement holds even in cases where no dental abnormalities are detected on their CT scans.^18^ The lack of unified diagnostic criteria is the most important reason for the misdiagonosis of OMS. In various researches at present, different diagnostic criteria for OMS are applied according to respective experience or needs. It not only leads to the misdiagnosis of OMS, but also obtains the different incidence of OMS in population, and also affects the diagnosis and treatment of OMS. Finally, a well-recognized management protocol has not yet been established for the diagnosis and treatment of OMS. Dental surgery, along with endoscopic sinus surgery (ESS) have been shown to yield superior outcomes.^19^ But the ideal sequence and timing are still controversial. Therefore, uniform diagnosis and managment standard and well-designed evidence-based studies are necessary for the clinical decision making and experimental research for OMS.

This present expert consensus will summarize the most recent development about the etiology, pathology, diagnosis and managment of OMS. Treatment strategy regarding different clinical scenarios will be directed by current literatures and discussed by multidisciplinary collaboration of otorhinolaryngologists and dental specialists. Some suggestions in this consensus come from published data. When no evidence to refer to, recommendations come from experts’ experience and opinion after discussion. At the same time, problems that need further investigation will be pointed out and discussed.

## Anatomy and biological fundamentals of maxillary sinus

The maxillofacial region encompasses four bilateral paranasal sinuses: the maxillary, ethmoid, frontal, and sphenoid sinuses. All paranasal sinuses are air-filled, mucosa-lined spaces and communicate with the nasal cavity through the sinus ostium. The maxillary sinus lies within the maxillary bone and is the largest and earliest one to develop. It is mature at the age of 12−14, with an average volume of about 15−20 mL.^4^ The morphology of the maxillary sinus is gradually developed throughout the growth process, from the initial elliptical structure to the pyramidal in shape at full maturity.

**Fig. 1:**
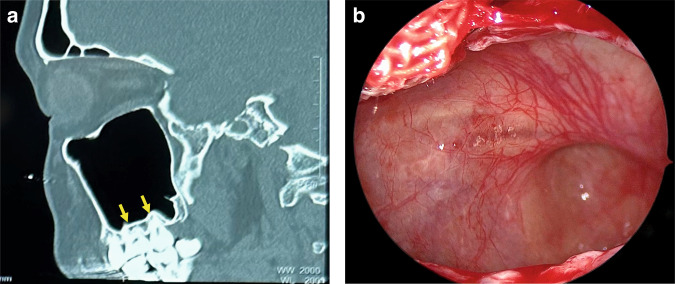
The relationship between the maxillary sinus floor and upper teeth. **a** CT image shows the roots of maxillary molars are located closest to the maxillary sinus floor. **b** Endoscopic image shows the maxillary sinus floor protrudes toward the oral cavity

The floor of maxillary sinus corresponds to the alveolar processes (Fig. 1a, b). The second molar roots are situated nearest to the sinus floor, with the first molar, third molar, second premolar, and first premolar sequentially positioned in relative proximity.^20,21^

**Fig. 2:**
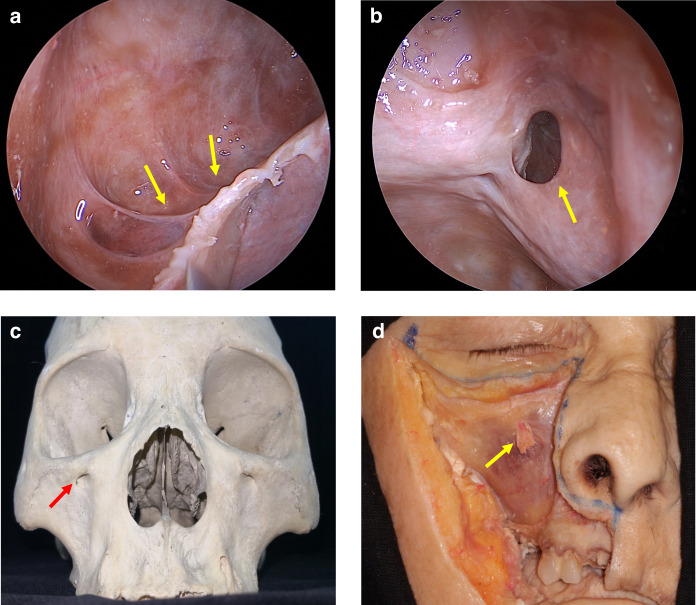
Anatomy and biological fundamentals of maxillary sinus. **a** Endoscopic image shows the alveolar recess. **b** Endoscopic observation of the natural opening in maxillary sinus. **c** The maxilla bone and the infraorbital foramen. **d** Anterior wall of the maxillary sinus and the infraorbital foramen (arrow indicates infraorbital nerve vascular bundle)

**Fig. 3:**
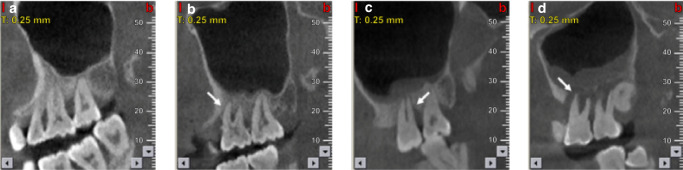
Cone beam computed tomography (CBCT) images of maxillary sinus mucosa.^25^ **a** Normal mucosa in patients with periodontitis. **b** Mild mucosal thickness (MT), left maxillary sinus (28-year-old woman, with furcation lesion of Tooth 26). **c** Moderate MT, left maxillary sinus 41-year-old man, with a vertical infra-bony pocket of Tooth 26 and peak-type MT. **d** Severe MT, left maxillary sinus (32-year-old man, with the sinus floor gap penetrated by inflammation caused by periodontitis)

**Fig. 4:**
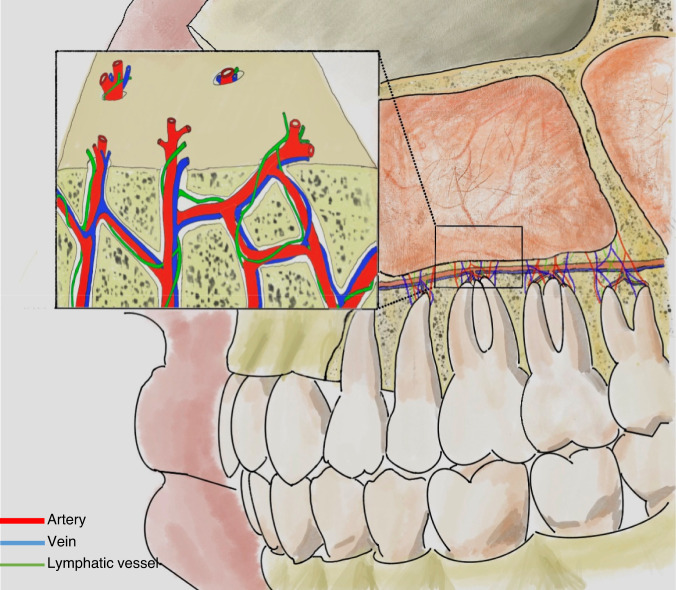
Vascular and lymphatic communication between maxillary sinus and teeth

As individuals age, the maxillary alveolar bone diminishes in thickness, resulting in a delicate mucoperiosteal layer that forms a barrier between the maxillary sinus and the oral cavity, known as the Schneiderian membrane.^22^ Some of the dental nerves, lymphatics and vascular plexus that supply the dental roots are located directly under the maxillary sinus mucosa.^23^ When the gasification of the sinuses is obvious, the third molar, premolar and canine may project into the maxillary sinus and be separated only by thin bone or mucosa, forming an alveolar recess (Fig. 2a). Anatomical structures above are potential risk factors for OMS.^24^ In normal conditions, the thick cortical of maxillary sinus floor can prevent inflammation of the dental roots from entering the maxillary sinus. When there is periapical lesions or severe periodontitis in upper dentition, the maxillary sinus mucosa has a tendency to thicken (Fig. 3),^25,26^ and the inflammatory mediators can even spread into the sinus cavity through the blood vessels, lymphatics and even muscle space.^19,27^ (Fig. 4).

**Fig. 5:**
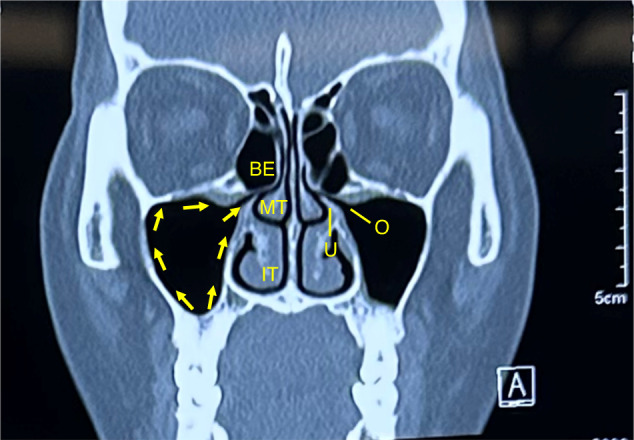
The drainage pathway of the maxillary sinus from the sinus floor towards the natural ostium into the middle meatus. *IT* inferior turbinate, *MT* middle turbinate, *BE* bullar ethmoid, *U* ucinate process, *O* ostium

**Fig. 6:**
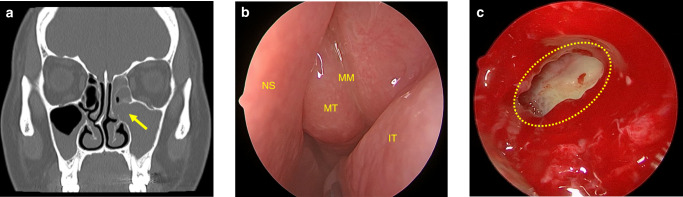
Maxillary sinus ostium obstruction caused sinusitis. **a** CT image shows maxillary sinus ostium obstruction (arrow) caused sinusitis. **b** Significant swelling of the mucosa of the middle turbinate and middle meatus can be observed. **c** The yellow circle points to the ostium and pus can be seen in the maxillary sinus in endoscopic operation. *IT* inferior turbinate, *MT* middle turbinate, *MM* middle meatus; *NS* nasal septum

The medial wall of the maxillary sinus simultaneously participates in forming the lateral wall of the nasal cavity, and the natural ostium of the maxillary sinus is located superiorly in the medial wall (Fig. 2b). The ostiomeatal complex (OMC) is an important concept in sinus surgery and represents the final drainage channel for the ethmoidal, maxillary and frontal sinuses (Fig. 5). Obstructive inflammation in this area will lead to mucosal swelling that affects the drainage of paranasal sinuses above, finally leading to sinusitis (Fig. 6).

The anterior wall is covered with skin and subcutaneous tissue, in which central inferior area is the weakest area (i.e.,Canine fossa) and can be removed into the maxillary sinus (i.e., Caldwell-Luc approach). The infraorbital foramen, which locates below the midpoint of the orbital rim, represents a critical anatomical feature of the anterior wall. This anatomical structure serves as the origin for the infraorbital neurovascular bundle. (Fig. 2c, d).

The superior wall of the maxillary sinus forms most of the orbital floor, separating the maxillary sinus from the orbital content. The infraorbital nerve vascular bundle passes through the infraorbital canal anteriorly, then come out of the infraorbital foramen and distributes in maxillofacial region (Fig. 2d).

The posterolateral boundary of the maxillary sinus constitutes the anterior barrier of both the pterygopalatine and infratemporal fossa. The thickness of posterolateral wall is different related to the degree of gasification of the maxillary sinus. The pterygopalatine process of sphenoid bone and the vertical plate of palatine bone are closely attached to it. Maxillary tubercle lies in the lower part of posterolateral wall, which is attached by the inferior head of lateral pterygoid muscle. Tumors within the maxillary sinus or in the pterygopalatine fossa region could invade bone of posterior wall.

## Etiology of Oms

**Fig. 7:**
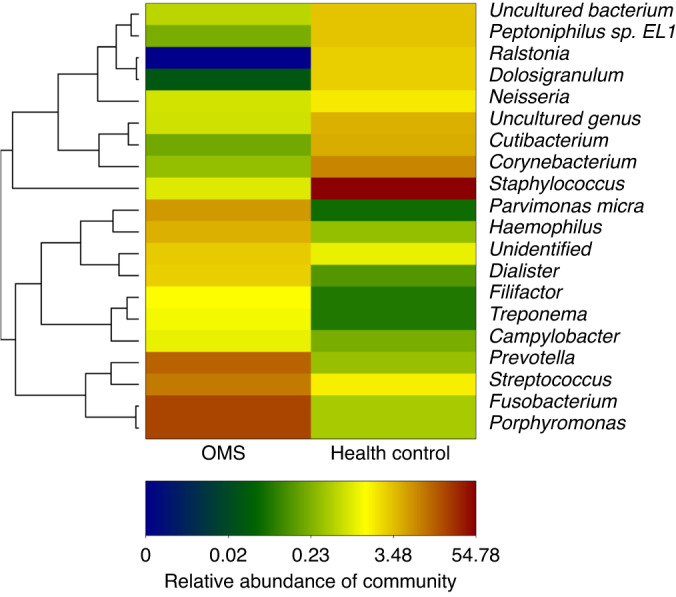
Relative abundance of genera from nasal secretions of OMS and control (people with simple nasal septum deviation). (*Porphyromonas, Fusobacterium*, *Streptococcus* and *Prevotella* were more abundant in OMS than control)

OMS is an infectious disease with definite oral infectious pathogeny. The microbiome plays a crucial role in OMS, exhibiting distinct characteristics compared to other types of rhinosinusitis.^28,29^ The OMS microbiome is generally predominated by anaerobic species from oral cavity and upper respiratory tract.^4^ Wu et al.^30^ showed that the alpha diversities of the microbiome in OMS patients were higher than that in controls (nasal septum deviation patients and impacted teeth extraction patients). The presence of a diverse microbial community suggested the coexistence of multiple pathogens within the ecosystem. In addition, the disparity of microbial structures between OMS patients and nasal septum deviation patients also indicates that the disruption of nasal microbiome, caused by odontogenic infection-induced microecological imbalance, necessitates individualized treatment approaches. In OMS patients, the nasal microbiome is predominantly comprised of anaerobic bacteria, including *Fusobacterium*, *Prevotella*, and *Porphyromonas* (Fig. 7). Several studies have documented a higher prevalence of these bacterial genera in OMS patients compared to chronic rhinosinusitis (CRS).^31–36^ In some other research projects, *Peptostreptococcus* was also detected.^28,31,32,35,37,38^ Colonization of these anaerobic bacteria are likely attributed by bacterial translocation from odontogenic infection.^35,39^

The detection of anaerobic bacteria within the nasal microbiome of individuals with OMS could suggest the occurrence of tissue hypoxia, or imply that the unique microenvironment presents in the mucus, or that the bacterial biofilms of OMS patients may be characterized by limited oxygen availability, thereby providing a niche for the survival of anaerobic bacteria.^40^

## Pathology of Oms

### Immunologic characteristics of OMS

Recent research progress has enhanced the acknowledgment of the discrete yet intersecting endotypes of CRS, delineated by inflammatory cytokine profiles of T helper 1 (Th1), Th2, Th17, and innate immune response pathways.^41,42^ However, the specific endotype of OMS remains understudied, with only two studies conducted to date. In a study based on patients from China, the inflammatory pattern analysis revealed that OMS exhibited lymphocyte and plasma cell-dominant cellular phenotypes, with Interleukin-17 (IL-17) being the dominant cytokine compared to interferon-γ (IFN-γ) and IL-5.^43^ Conversely, the other study based on patients from USA reported that OMS had significantly elevated levels of Th1 markers (IFN-γ, tumor necrosis factor-α) and innate immune markers (IL-6, 8, 10, 27, and C-X-C motif chemokine ligand 9) compared to healthy controls.^44^ Notably, the levels of IL-17 were similar in both OMS patients and controls.^44^ Owing to the constrained case count in both investigations, the true immunologic mechanism of OMS remains unclear. Further in-depth investigations are warranted to gain a deeper understanding of the immunologic pathways involved in OMS.

### Histopathology characteristics of OMS

**Fig. 8:**
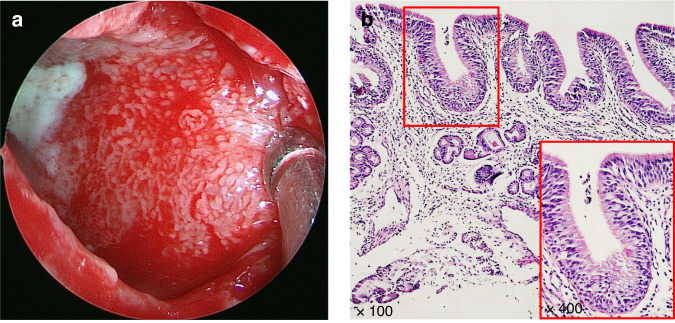
Formation of the papillary-like folds in maxillary sinus mucosa in OMS.^43^ **a** Endoscopic image of the maxillary sinus mucosa of an OMS patient showing purulent secretion, diffuse edema and remarkably small papillary protrusions. **b** Tissue section stained by HE demonstrating that a type of papillary-like fold was found in all the maxillary sinus mucosa samples of the OS patients, as well as that the mucosa was still covered with intact pseudostratified columnar ciliated epithelium

The histopathological features of the maxillary sinus mucosa in patients with OMS are distinctive. The normal nasal sinuses are lined with a pseudostratified ciliated columnar epithelium. Through mucociliary clearance of the epithelium, inhaled pollutants, allergens and pathogens can be excluded,^27^ which is an important defense mechanism.^20^ For OMS, its maxillary sinus mucosa shows a gyrus-like appearance, presenting papillary folds which is covered by a complete pseudolamellar columnar ciliated epithelium (Fig. 8).^43,45–47^ Furthermore, the secretions of OMS are notably purulent rather than viscous, but the number of ciliated epithelial cells remaines unchanged and the goblet cells do not exhibit hypertrophy.^37,48^ These observations suggest that the ciliated columnar epithelium undergoes no substantial damage and no irreversible damage occurres. Moreover, the epithelium of OMS consistently shows elevated expression levels of claudin-4 compared to those observed in chronic rhinosinusitis without nasal polyps (CRSsNP), chronic rhinosinusitis with nasal polyps (CRSwNP), and control subjects, implying a potential enhancement of epithelial conjunction in OMS.^43^

However, the interplay between ciliary mucosal function and bacterial infections, and ostiomeatal complex occlusion may lead to closure of the vicious cycle of maxillary sinus inflammation, finally culminating in intractable OMS.^49^ The inflammatory process of OMS can be delineated into two different stages: an acute or invasive stage marked by the innate immune defense activation, predominantly featured by neutrophils and macrophages, followed by a chronic stage, during which the lesion exhibits characteristics of an adaptive immune reaction. During the acute phase, bacteria can pervade adjacent tissues directly, stimulating the membrane epithelium and inciting a hypertrophic reaction.^50^ In addition, bacteria from tooth pathological processes, via microbial toxins, can amplify inflammatory mediators and potentially induce alterations in ciliary activity.

Zhang et al.^43^ reported that, in 25 OMS patients, nasal polyps were absent in 88.5% of them, while 8.2% had polyps limited to the middle nasal meatus, and 3.3% had polyps outside the middle nasal meatus. Raman et al.^48^ suggested that OMS histopathology was more similar to CRSsNP with more severe inflammation.^51^ Compared to CRSsNP, OMS had an increased incidence of moderate to severe inflammation. Another study showed that about 80% patients with OMS had severe chronic inflammation.^39^ In contrast to CRSwNP, the main inflammatory cells in chronic OMS were lymphocytes and plasma cells,^39,48^ and varying degrees of eosinophils and neutrophils were shown in certain tissues but these cells never dominated lymphoplasmic cells.^39^ Moreover, squamous metaplasia and fibrosis were reduced in OMS, and some eosinophiliosis was shown, but to a lesser extent, compared to CRSwNP.^48^

However, the originatation of the inflamed epithelium in OMS remains unclear. It could potentially stem from the epithelium within the odontogenic lesion, which subsequently spreads into the sinus cavity and envelops the lesion. Alternatively, it could originate from the sinus epithelium, differentiating under the influence of the underlying capsular connective tissue, which is fundamentally a derivative of the periodontal ligament.^49^

## Clinical features and symptoms of Oms

**Fig. 9:**
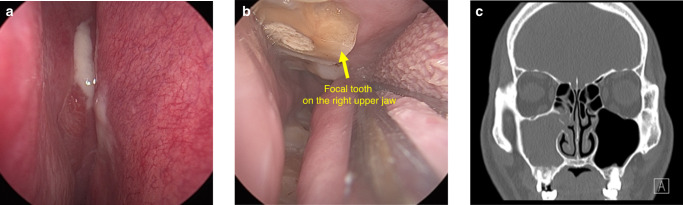
The clinical feature of a typical OMS patient. **a** Purulent secretion with mucosal swelling and congestion in the right middle nasal meatus under endoscope. **b** The focal tooth on the right upper jaw under endoscope. **c** The root of focal tooth protruding into the floor of the maxillary sinus with local discontinuous bone erosion on CT

OMS originates from dental sources, and occurs firslty in the floor of the maxillary sinus as a result of periodontitis, AP, oroantral fistulas, or as a sequel to complications arising from dental procedures. Recent insights, as outlined in the European Position Paper on Rhinosinusitis and Nasal Polyps 2020 (EPOS), have established that OMS should be regarded as a separate entity from non-odontogenic maxillary sinusitis (non-odontogenic MS),^1^ which is categorized as a unilateral secondary chronic sinusitis attributed to localized dental lesions.^52^ There are different kinds of clinical features between OMS and non-odontogenic MS.^7,53,54^ Thereinto, the foul smell and head and facial pain are especially familiar in the former, but are extremely rare in the later. Certain characteristics are notably linked with OMS. These include a malodorous scent, unilateral facial pressure, and pus at the middle meatus accompanied by regional mucosal hyperemia and swelling under endoscope. Additionally, opacification of the sinus revealed by CT, was also identified as a significant indicator of OMS.^17,55^ Additionally, compared to non-odontogenic MS, OMS patients often have a history of toothache, tooth looseness, or gingival abscess on the affected side of maxillay sinus. (Fig. 9) By oral clinical examination, tooth decay, tooth percussion pain, or gum redness and swelling may be found. In addition to maxillary sinus inflammation, OMS can cause serious complications in some extreme cases. When the inflammation of OMS spreads, the infection can spread from the maxillary sinus to the facial soft tissues, periorbital, intraorbital, and even into the cavernous sinus or intracranial region, thus leading to facial cellulitis, orbital cellulitis, orbital abscess and cavernous sinus thrombophlebitis or intracranial infection.^56,57^

## Classification and diagnosis of Oms

### Classification of OMS

According to disease progression, OMS can be divided into acute OMS and chronic OMS. Fever, headache, suborbital pain, nasal congestion and runny nose, with or without post-nasal discharge are common symptoms of acute OMS.^3^ If disease is not controlled in the acute phase, acute OMS can develop into chronic OMS. At this stage, fever, headache, and suborbital pain could be alleviated, but nasal symptoms such as nasal congestion and runny nose often continue. In general, the symptoms of OMS are similar to that of MS, but the pain and foul smell are normally more pronounced in the former. It is worth noting that symptoms above may not always occur, due to the opening of OMC. When the OMC is opening, odontogenic infectious substances could flow out through the maxillary sinuses.

According to different origin, the dental source of OMS can be classified into four categories: *Pulpal origin*, including pulp necrosis, periapical inflammation, root fracture and other kinds of endodontic infection. *Periodontal origin*, referring to periodontal defect with severe alveolar bone absorption (more than two-third of the root length^56^). *Pulpal-periodontal origin*, referring to involved diseased tooth with periodontal-endodontic combined infection. *Other origins*, referring to oroantral fistula or foreign objects forced into the sinus during tooth extraction or other oral surgeries.

### Diagnostic criteria of OMS

Diagnostic criteria for OMS are currently heterogeneous, and different scholars hold different opinions.^58^ Imaging is an indispensable means to diagnose OMS, but it can never be the only evidence. In a healthy state, maxillary sinus radiographically appears as translucent and well-defined cavities. While in a state of illness, image of sinus presents thickened mucosa, air-fluid level, or opacification. It is generally accepted that the sinus mucosa with thickness more than 2 mm is pathologically significant.^59–61^ But patients with only mucosal thickeness should not be diagnosed with OMS. In some situation, the opacified maxillary sinus, air-fluid level or the thickened sinus mucosa may even coexists with odontogenic lesions, but neither can we jump into a conclusion of OMS easily. In order to diagnose OMS definitely, in addition to the lesion of maxillary sinus, it is necessary to identify the odontogenic lesions, and confirm the exact correlation between maxillary sinus lesion and oral lesion.

Maillet^60^ recommended a soft-tissue density mass within the sinuses was odontogenic origin if it fulfilled the criteria: *carious tooth, tooth with defective restoration or extraction site, with or without radiographically evident periapical lesion, and mucosal thickeness was limited to the area of the tooth or extraction site*. In 2018, more rigorous and detailed criteria was proposed by Ly.^62^ She supported that, with predominantly unilateral sinus opacification on CT, OMS would be diagnosed as follows: *An oral lesion associated with the affected sinuses is ensured. Patients have a history of dental disease or dental treatment of the upper dentition on the same side of MS in temporal relation to the symptom onset and CT finding. There are radiological signs of a dental abscess or an oral-antral communication*. These diagnostic criteria have been further simplified by Yoshida^63^ with sinus CT shows: *Apical root lesion in a maxillary tooth. Maxillary sinus opacification. Maxillary bone defect between the maxillary sinus floor and periapical root*. However, in these current criteria, only the pulpal source of oral infection was considered but the periodontal infection was ignored. In terms of this problem, some studies now began to focus on the influence of periodontal infection in OMS.^55^ In this consensus, we summarized and proposed an improved diagnostic criteria of OMS based on: *1.Patients with clinical symptoms of MS, with or without oral symptoms. 2.There are sick teeth in the upper dentition on the same side of MS, with periapical lesions or severe alveolar bone loss (absorption to2/3 of the root or more)*^56^ *on CBCT. 3. There is foreign body in the maxillary sinus or ipsilateral oroantral fistula on the same side of MS. 4. CT/CBCT images present air-fluid level or maxillary sinus opacification or thickened maxillary sinus mucosa(>2* *mm)*^59,60^ *limited to ipsilateral oral lesion on the same side of MS, with feature of: a. discontinuity of maxillary sinus floor between them. b. a thin layer of floor bone remains between them. c. a thick floor bone between them*.

**Fig. 10:**
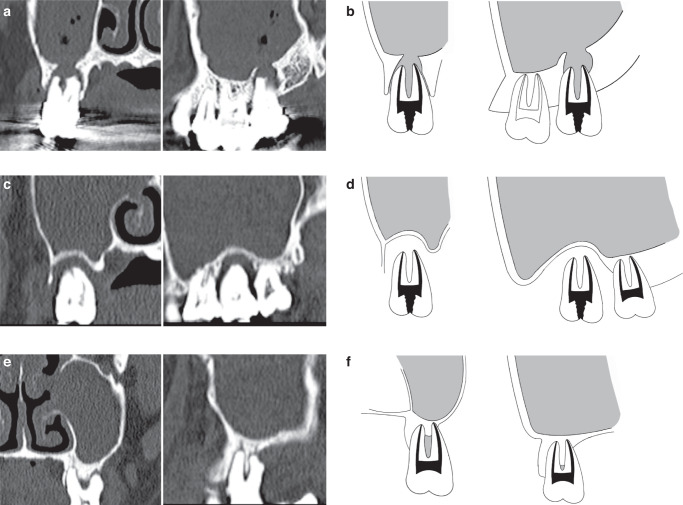
Representative sinus CT images of OMS. **a**, **b** Definite evidence: CT images show the discontinuity of maxillary sinus floor in the site of dental lesion. **c**, **d** Potential evidence: CT images show a thin layer of floor bone remaining between the maxillary sinus floor and oral lesion. **e**, **f** Questionable evidence: CT images show a thick layer of floor bone remaining between the maxillary sinus floor and oral lesion

We classify these proofs supporting OMS into A, B and C three categories (Fig. 10):

A. *Definite evidence: Patients who meet the criteria 1, 3 or 1,2, 4a*.
B. *Potential evidence: Patients who meet the criteria 1,2, 4b*.
C. *Questionable evidence: Patients who meet the criteria 1,2, 4c*.

### Differential diagnosis of oms and non-odontogenic MS

OMS and non-odontogenic MS are sometimes difficult to distinguish. Based on clinic symptoms and previous diagnosis and treatment history patients described, we can get an initial impression. For non-odontogenic MS patients, inflammation can be found in unilateral or bilateral maxillary sinus, and bilateral is more common, but OMS patients usually present inflammation in unilateral maxillary sinus.^52^ In a retrospective analysis of 121 OMS cases who received surgical intervention, 92.6% were found to be unilateral and only 7.4% were bilateral.^64^ Besides, Matsumoto^9^ reported that 72.6% of the 190 cases of unilateral sinusitis were OMS. According to a meta-analysis assessing 12 studies, 50% of unilateral MS were reported to be OMS.^11^ In addition to unilateral maxillary sinus involvement, it should be noted that some symptoms, such as foul smell, ipsilateral facial pressure,and middle meatal pus, may be more specific for OMS.^3,31^

Toothache sometimes happens in patients with OMS. Their toothache can be caused by pulp nerve exposure, the spread of odontogenic infection to periapical tissue, or periodontal inflammation. In general, this kind of pain is characteristically localized and highly related to temperature stimulation and occlusion. Non-odontogenic MS can also cause toothache, which usually radiates to all (posterior) teeth on the same side of the upper dentition. These teeth are sensitive to percussion, but their pulp vitality is normal. Therefore, in order to distinguish OMS from non-odontogenic MS, otolaryngologists should pay more attention to the past oral history of MS patients and refer to dentist for careful oral examination if necessary. A detailed dental examination will help us to determine whether the oral lesion is a cause. The specific teeth examination includes steps as follows: inspection of buccal mucosa and oral vestibule for the mucosal swelling or erythema, percussion for the teeth pain, electric or thermal pulp testing for the vitality of teeth, and probe for deep periodontal pockets. We would discover that OMS patients have one or more dental problems, including periapical lesions, severe periodontitis, or even oroantral fistula, on the same side of MS.

After clinical examination, OMS can be finally definitely diagnosed by means of imaging examination. CT and CBCT are common methods applied to diagnose OMS. CT, displaying both soft tissue and bone tissue in three-dimensional way, can identify oral lesions and maxillary sinus clearly, and locate the foreign bodies in maxillary sinus clearly.^65^ But many dental lesions in detail are difficult to identify on CT.^17,66^ By contrast, CBCT, which has higher sensitivity than CT for identifying odontogenic lesion and the relationship between the lesion and the maxillary sinus floor, is now widely used in OMS diagnosis.^67,68^ But it has limitations in displaying the full view of the maxillary sinus. So in clinical practice, both CBCT and CT are applied based on specific needs.

**Fig. 11:**
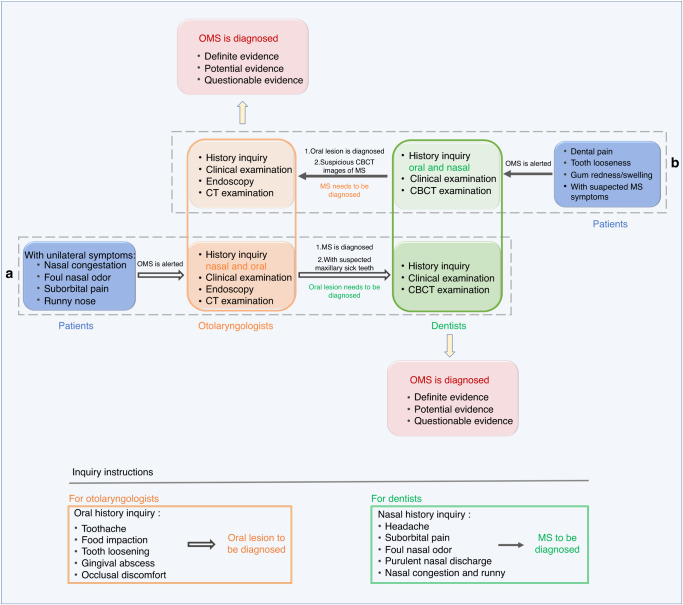
The standard flowchart for OMS diagnosis and treatment in the clinical treatment process. *MS* maxillary sinusitis, *OMS* odontogenic maxillary sinusitis, *ESS* endoscopic sinus surgery

In conclusion, the combination of clinic symptoms, oral and nasal examination, and radiological imaging will help us differentiate OMS from non-odontogenic MS. To be specific, when a patient coming to rhinologists presents with unilateral MS, especially with unilateral nasal purulent discharge and foul smell, OMS should be alerted. Next, we should confirm whether the patients ever have ipsilateral maxillary dental problems, with the help of dentists. A detailed oral examination by dentists is necessary for finding the suspicious ipsilateral pathogenic teeth. Then CT/CBCT examination can ultimately clarify the correlation between the oral lesion and the maxillary sinus, and enable us to make a definite diagnosis of OMS (Fig. 11a). In rare cases, some OMS patients seek treatment firstly in dental clinics. When dentists find patients with suspicious symptoms of MS, accompanied by suspicious imaging features of MS on the same side of oral lesion, we should refer the patient to the rhinologic department for the definite diagnosis of MS. Then after clarifying the relationship between the imaging of MS and that of the oral lesion, we can finally confirm the diagnosis of OMS (Fig. 11b).

## Treatment principles of Oms

Because of the unique etiological and pathological characteristics of OMS, its treatment is different from non-odontogenic MS and involves the cooperation of rhinologists and dentists. The nasal treatments include non-surgical therapy and ESS surgical intervention, aiming to promote the resolution of inflammation, correct the obstruction of maxillary sinus natural ostium, remove irreversible lesions, to improve the symptoms of patients. The dental treatments mainly include root canal treatment, periodontal treatment, apical surgery, focal teeth extraction, and fistulas repair to remove odontogenic infection and avoid recurrence of OMS.

### Treatment of maxillary sinus lesions—Rhinological treatment

#### Non-surgical treatment

Non-surgical treatment is the first and most important step, including antibiotic therapy, nasal corticosteroids and nasal irrigation, which helps to improve the symptoms of patients. Compared to non-odontogenic MS, OMS with a significant bacterial infection, so antibiotic therapy is an important option.^36^ The use of penicillin (amoxicillin) and β-lactamase inhibitors, with or without metronidazole, can generally fight a wide range of multi-microbial and anaerobic populations.^53,69^ It should be noted that the antimicrobial therapy should be guided by the antibiotic resistance pattern. Zirk et al.^64^ conducted a study on 121 patients with OMS and found that piperacillin/tazobactam (93.9%), cotrimoxazole (83.3%), ampicillin/sulbactam (80%), cefotaxime (78.1%), cefuroxime (69.4%), ampicillin (68%), and clindamycin (50%) had the highest sensitivity, and for patients with confirmed penicillin allergy, fluoroquinolones such as moxifloxacin (86.2%) and ciprofloxacin (62.2%) and tetracycline (62.9%) can be used as alternatives. Saibene et al.^36^ showed that among the isolated bacteria strain from 28 OMS patients, 70% were sensitive to amoxicillin, and all species of the isolated bacteria strain were sensitive to the combination of levofloxacin, teicoplanin, and vancomycin. However, existing studies have shown that antibiotic therapy alone is difficult to cure OMS.^70,71^

#### Surgical treatment

Surgical treatment is advised if conservative treatment is ineffective. In the past, the classical Caldwell-Luc procedure was the main surgical management of maxillary sinus disease, but with the disadvantages of large trauma and many complications. At present, ESS is regarded as the gold surgical standard which is an alternative to Caldwell-lLuc approach,^1^ and has shown excellent effect, especially when the OMC is blocked. On one hand, ESS provides a large middle meatal antrostomy. On the other hand, it improves visualization for the entire maxillary sinus through a smaller surgical window. Thanks to the small surgical trauma and optimal exposure, ESS could maximumly eliminate infection of maxillary sinus by opening OMC and removing sinus lesion to restore normal drainage and ventilation while keep the healthy mucosa undamaged, thereby reducing possible complications. ESS is often required for OMS, especially for intractable OMS.^72^

### Treatment of oral lesions – dental treatment

#### Tooth preservation

Root canal therapy, apical surgery and periodontal therapy can be performed when the focal tooth is evaluated to be excellent/good/fair, or questionable but infection could be controlled, or patients have strong desire to preserve tooth.^18^ A recent cohort study showed that 13% (9/68) OMS patients improved after conservative dental treatment.^73^

#### Tooth extraction

The focal tooth can be extracted when it is evaluated to be hopeless (prognosis) or questionable but the infection is difficult to control. Simuntis et al.^74^ published a prospective study on 96 patients with OMS due to AP, and they demonstrated a 77% success rate with dental extraction alone.^74^ However, a study on 37 patients with OMS found that even after tooth extraction, OMS may not improve, particularly in younger patients.^75^

## Multi-disciplinary treatment sequence of Oms

The optimal sequence of nasal and dental treatments for OMS has not still been identified.^19,76,77^ Many studies recommended that dental treatment to eliminate the source of infection should be done before nasal treatment.^53,78^ Longhini and Ferguson^78^ suggested that dental treatment should precede ESS, since they found that without dental intervention, OMS can be not cured by ESS alone. And Yoo et al.^79^ even reported that 67% of 33 OMS patients were recovered after medical and dental treatment, and only 33% of the patients required additional ESS. On the contrary, some other authors recommended ESS in priority. Abdulkader^80^ conducted a retrospective cohort study which demonstrated that ESS followed by dental treatment resulted in a shorter treatment period than dental treatment followed by ESS. There is no doubt that both rhinologolical and dental treatment are necessary,^81–83^ although the ideal sequence of management has not been presented yet.

**Fig. 12:**
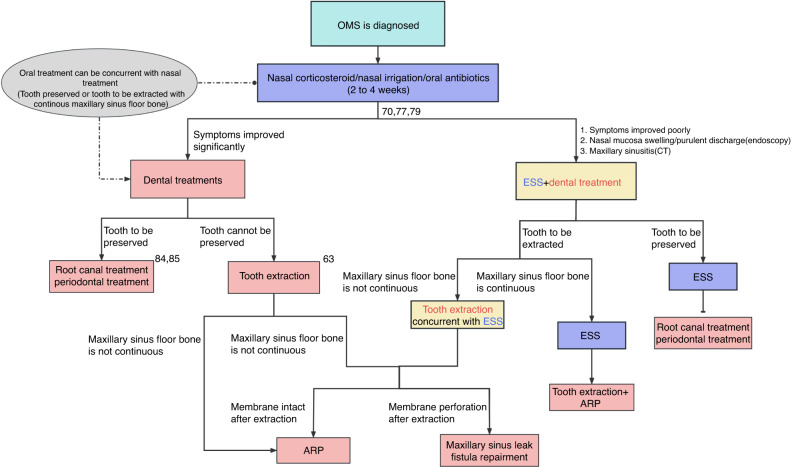
Decision-making tree for OMS management in clinical practice.^63,70,77,79,84,85^ *OMS* odontogenic maxillary sinusitis, *ESS* endoscopic sinus surgery, *ARP* alveolar ridge preservation

We thus suggest that the ideal treatment sequence for OMS should depend on individual conditions of patients, in the context of the latest diagnostic criteria. Based on systematically reviewed literature and practical experiences of expert members, the evidence and consensus-based clinical practice guideline for the management of OMS is formed as follows (Fig. 12)^63,70,77,79,84,85^:

### Rhinological non-surgical treatment for 2−4 weeks for OMS

Non-surgical treatment is the primary method to control the acute symptoms of OMS. Rhinological non-surgical treatments, such as antibiotics, corticosteroid and irrigation, should be done firstly, during which, dental treatment could be done depending on the oral lesion involved.

### Dental treatment with no rhinological surgical treatment for OMS

Dental treatment with no rhinological surgical treatment is suggested, if the disease burden imparted by sinusitis (based on symptoms, endoscopy or CT) is low after rhinological non-surgical treatment.

#### The focal teeth to be preserved

When teeth are evaluated to be saved by dentists, corresponding dental treatments should be done.

#### The focal teeth to be extracted

Teeth with no hope of preservation should be extracted. And following dental surgeries will applied depending on different intra-operative findings:

***Maxillary sinus floor Bone is continuous*** *.*

In this situation, (alveolar ridge preservation, ARP) is recommended after teeth extraction.

***Maxillary sinus floor Bone is not continuous*** *.*

If maxillary sinus mucosa is intact after extraction (no communication between oral cavity and maxillary sinus), ARP is recommended after teeth extraction.

If there is membrane perforation (communication between oral cavity and maxillary sinus), simultaneous or delayed maxillary sinus leak and fistula repairment are recommended after extraction.

### Dental treatment combines with rhinological surgical treatment for OMS

Contemporaneous or non-contemporaneous dental treatment and rhinological surgical treatment are suggested, if the disease burden imparted by sinusitis (based on symptoms, endoscopy or CT) is heavy and OMC is blocked, after non-surgical treatment.

#### The focal teeth to be preserved

ESS should be done firstly, and then corresponding dental treatments should be done for focal teeth to avoid recurrence of OMS.

#### The focal teeth to be extracted

Teeth with no hope of preservation should be extracted during or after ESS operation, according to following situations:

***Maxillary sinus floor Bone is continuous*** *.*

Teeth extraction and ARP after ESS operation are recommended.

***Maxillary sinus floor Bone is not coninuous or latrogenic OMS*** *.*

Teeth extraction during ESS operation is recommended, and following dental surgeries will applied depending on different intra-operative findings:

If maxillary sinus mucosa is intact after extraction or removal (no communication. between oral cavity and maxillary sinus), ARP is recommended during operation.

If there is membrane perforation (communication between oral cavity and maxillary sinus), simultaneous or delayed maxillary sinus leak and fistula repairment are recommended. ESS combines with oroantral fistula closure could resolve 90%-100% of OMS cases.^70^

To sum up, depending on patients’ condition and considering the risks, benefits, period, and costs of treatments, rhinologists and dentists make the optimal therapeutic decision with various combinations of medication, ESS, and dental treatment.

### The outcome of OMS after treatment

There is now a lack of systematic evaluation of the prognosis of OMS treatment. Felisati et al.^82^ introduced that OMS patients could be treated with the success rate of 99% (254/257) by ESS combining with dental treatment. And in the study of Wang et al.^19^ among the 21 recovered patients, 33% (7) were resolved by ESS alone, 33% (7) were resolved by ESS combined with dental treatment, and 10% (2) were resolved by dental treatment alone. However, it has also been reported that 29% of OMS patients were refractory to ESS alone.^78^ The large differences between results of these researhces above may be related to different patient volumes, different inclusion criteria for OMS, and the severity discrepancy of the dental disease and sinusitis. For example, OMS patients who recovered after dental treatment alone may have a low sinus disease burden, whereas those who suffering from mild dental disease may completely recover after ESS alone.^53,86^ We suggest that the prognosis of OMS depends on the precise underlying diagnosis of the etiology and corresponding individualized treatment. Usually, OMS with a clear etiology will not recur after eliminating oral lesions or combining oral and nasal treatments. However, some factors absolutely affect the prognosis of OMS. Sakkas A et al.^87^ analyzed the prognosis of 164 OMS patients and found that patients with a history of antiresorptive-related osteonecrosis of the jaw showed a significant tendency toward disease recurrence, and that age, disease site, surgical approach and surgical method were not associated with OMS recurrence.

## Complications and management of Oms operation

### Complications and management of ESS

Based on the degree of odontogenic infection, ESS procedure could extend from solo maxillary sinus to ethmoid sinus, even sphenoid sinus and frontal sinus, by employing the visualization of rigid 0 degree angular endoscope and other surgical instruments. Besides, there are some important anatomical location adjacent sinuses, like the orbit, optic nerve, skull base, internal carotid artery and anterior ethmoidal artery, etc. So all types of ESS procedures are inclined to potential risks and complications, but serious complications are fortunately rare. A study involving 50734 patients undergoing ESS from 706 hospitals reported that the major complication rate of ESS was about 0.5%, which consisted of 0.09% cerebrospinal fluid leakage, 0.09% orbital injury, 0.1% hemorrhage requiring surgery, and 0.18% hemorrhage requiring blood transfusion.^88^ There are some measures to reduce the incidence of these complications. First of all, it must be kept in mind that any ESS procedure should only be performed after appropriate training and adequate understanding of important anatomical landmarks and sophisticated variations. Secondly, careful review of preoperative image data could make surgeons identify the safe boundaries and possible anatomical defect in advance to avoid the injury to important nerves and vessels, as well as the orbi.^89^ Thirdly, although the emergence of powered microdebriders is the revolutionary breakthrough in ESS, inexperienced surgeons should be prohibited to use them nearby the orbital cardboard area, in order to prevent irreversible damage to the intra-orbital fat and medial rectus muscle. Fourthly, positioning patients in 15 degrees reverse trendelenburg position,^90^ application of topical vasoconstrictive agents,^91^ application of total intravenous anesthesia,^92^ and other efforts can be attempted to decrease intra-operative bleeding, improve the visualization during ESS, and avoid disaster effects. Last of all, the incidence of lacrimal duct injury (LDI) during maxillary antrostomy could reach 15%, since the lacrimal duct is adjacent to the uncinate process.^93^ Luckily, LDI does not always develop clinical symptoms. Therefore, when LDI happens, observation alone is a suitable therapeutic strategy in the postoperative several month.^93^ If needed, endoscopic dacryocystorhinostomy could treat the persistent epiphora secondary to LDI effectively.

### Complications and management of dental operation

There is no significant difference in the general management of dental treatment and related complications between OMS patients and patients with only oral diseases, but there are still some differences between them in detail.

#### Complication of tooth extraction

Oroantral communication and tooth displacement are common complications during tooth extraction for OMS patients, which should be paid attention to.

##### Oroantral communication

Periapical and periodontal lesions with severe local alveolar bone absorption in OMS patients can cause discontinuity or a thin layer of floor bone remaining between the maxillary sinus and oral lesion. In this situation, tooth extraction may cause oroantral communication. ESS combing with maxillary sinus leak and fistula repairment are recommended for oroantral communication. At the same time, antibiotics are also recommended for infection prevention.

##### Teeth roots displaced into the maxillary sinus

If there is discontinuity or a thin layer of floor bone between the maxillary sinus and oral lesion in OMS patients when teeth extraction, in addition to oroantral communication, the roots of teeth may be displaced into the maxillary sinus. If the roots do not completely enter into the sinus cavity, it can usually be found and removed under direct vision. If the roots have been completely displaced into the maxillary sinus, it needs to be removed by flap opration and bone removal.^94^ The application of endoscopic technology can greatly reduce the trauma,^95,96^ especially in the cases that the traditional window-opening irrigation fails or when foreign bodies adheres to the maxillary sinus mucosa. Hence, for OMS patients with teeth completely enter into the sinus who need ESS, teeth can be removed during surgery.

##### Infection after tooth extraction

The mostly common chronic infection after tooth extraction is caused by residual granulation tissue, tooth pieces, bone pieces, and calculus, but acute infection is also likely to occur in rare cases.^97^ The affected teeth of OMS patients ofen have serious infection, and the key point to prevent chronic infection is to perform debridement radically and apply antibiotics after tooth extraction.When infection is found, inflammatory granulation tissue and foreign bodies should be removed thoroughly under local anesthesia to promote extraction socket healing.

##### Post-operative bleeding

Bleeding after tooth extraction is often caused by improper nursing or local factors, and also by few systemic factors. When there is bleeding after tooth extraction, we should put emphases on the systemic situation of OMS patients firstly, and blood tests should be performed if necessary.^98^ Then, careful oral examination should be carried out to clarify the cause of bleeding. For bleeding caused by improper nursing, residual granulation tissue, soft tissue tear, and microvascular injury, measures such as appropriate nursing, debridement, compression, and suturation, in conjunction with packing materials like iodoform sponge and hemostatic gauze, can provide a good solution.^99^ However, for bleeding with systemic background, in addition to local hemostatic measures, systemic treatments like systemic administration and necessary blood transfusion are needed.

##### Dry socket

Dry socket (alveolar osteitis) is a complication of dental extraction and usually occurs in mandibular molars extraction. For OMS patients, the incidence rate of dry socket is relatively low. If happens, the socket should be debrided thoroughly till fresh blood apprears, and iodoform sponge can be used to promote healing.^100^

#### Complications of root canal treatment

Due to severe damage of the alveolar bone in OMS patients, root canal therapy may has a significant impact on the maxillary sinus, and root canal therapy should be performed more careful.

##### Post-operation pain

Post-operation pain is a common complication of root canal treatment. For cases with mild swelling and pain, painkillers can alleviate symptoms effectively. Sometimes, a high contact point also leads to pain and can be solved by means of occlusal adjustment. If the pain lasts for several days and the radiograph shows overfilling root canal materials, root canal retreatment should be considered after inflammation reduction.^101^ For cases with severe symptoms, such as abscess formation, cellulitis or even systemic symptoms, abscess incision and drainage need to be performed, and antibiotics or systemic support therapy should be considered if necessary.

##### Intracanal separated instruments in the root canal

For OMS patients, the measures for handling fractured instruments is no different from that in routine root canal treatment.^102^ But overfilling and fractured instruments outside the roots should be alerted. In this case, when patients with obvious symptoms of the maxillary sinus, root canal re-treatment or separated instruments removal (apical surgery or tooth extraction if necessary) should be conducted in time, if not, routine follow-up is needed.

#### Complications of apcial surgery

When removing the inflammatory tissue during apical surgery, surgeons should caution the damage to the maxillary sinus floor and avoid perforation.

##### Post-operative bleeding

Post-operative bleeding can be usually stopped by appropriate nursing, compression, and suturation, in conjunction with packing materials. For patients with systemic background, the management is the same as that of post-operative bleeding after tooth extraction.

##### Post-operative pain

Post-operative pain is generally mild, and non-narcotic analgesics can be considered.

##### Pos-toperative infection

For OMS patients, antibiotics should be routinely given after surgery.^103^ If infection happens, debridement and medication are good solutions.

#### Complications of periodontal treatment

Periodontal treatment rarely leads to serious complications of maxillary sinus when patients with no maxillary sinus floor destruction. However, for OMS patients with thin or discontinued maxillary sinus floor, attention should be paid to preventing perforation and avoiding pushing infection into the maxillary sinus during treatment.

##### Post-operative bleeding

When bleeding happens after periodontal initial treatment, subgingival calculus and infectious granulation tissue should be checked and debrided carefully firstly, then the pocket should be rinsed thoroughly with H_2_O_2_, and periodontal dressing could be applied when necessary.

When bleeding happens after periodontal surgical operation, the effective methods include appropriate nursing, compression and saturation, in conjunction with packing materials.

##### Post-operative periodontal abscess

When there is periodontal abscess, the pocket should be rinsed thoroughly with chlorhexidine, and local incision and antibiotics should be given when necessary.

##### Post-operative pain

Post-operative pain after periodontal treatment is generally mild, and non-narcotic analgesics can be considered.

##### Pos-toperative infection

Antibiotics should be routinely given after surgery.^104^ If infection happens, debridement and medication are good solutions.

## Conclusions and expectations

OMS is a common form of MS, with a clear etiology, pathology and relatively good prognosis. However, the actual incidence of OMS is still unknown and the diagnosis of OMS is underappreciated. Due to the interdisciplinary nature of this disease, rhinological or dental management alone often does not achieve the optimum treatment effect, so multi-discipline treatment is essential. Because of the diversity of odontogenic diseases and the various degree of sinus inflammation (from sinus mucosa thickening to extra sinus invasion), the ideal sequence or timing of management is still controversial. To address this question, well‐designed evidence-based studies collaborated by rhinologists and dental specialists are necessary for meaningful progress about the management strategy of OMS.
